# Molecular Characteristics and Treatment Implications of *TP53* Gain‐of‐Function Mutations in Non‐Small Cell Lung Cancer

**DOI:** 10.1002/cam4.71215

**Published:** 2025-10-23

**Authors:** Zheng Zhao, Rui Lu, Feng Zhang, Hanlin Chen, Qiuxiang Ou, Xiaotian Zhao, Xinyue Hong, Hua Bao, Degan Lu, Jie Min

**Affiliations:** ^1^ Department of Clinical Oncology Shaanxi Provincial Cancer Hospital Xi'an China; ^2^ Department of Thoracic Surgery Zhongnan Hospital of Wuhan University Wuhan China; ^3^ Department of Oncology Tangdu Hospital, the Fourth Military Medical University Xi'an China; ^4^ Geneseeq Research Institute Nanjing Geneseeq Technology Inc. Nanjing China; ^5^ Department of Respiratory Medicine The First Affiliated Hospital of Shandong First Medical University & Shandong Provincial Qianfoshan Hospital Jinan China

**Keywords:** immunotherapy, next‐generation sequencing, non‐small cell lung cancer, survival, *TP53* gain‐of‐function

## Abstract

**Background:**

*TP53* gain‐of‐function (GOF) effects lead to cellular responses beyond the capabilities of wild‐type *TP53* and are known to promote cancer progression, resulting in poorer outcomes in cancer.

**Methods:**

A total of 486 patients diagnosed with non‐small cell lung cancer (NSCLC) with baseline DNA sequencing data were enrolled in our study cohort. In addition, clinical and sequencing data from external NSCLC cohorts, including a cohort with histologic data (*N* = 219), a combined cohort from two studies treated with immunotherapy (*N* = 315), and a cohort treated with ROS1 tyrosine kinase inhibitor (TKI) (*N* = 50), were analyzed to assess the relationships between *TP53* GOF mutations and histologic subtypes, immunotherapy outcomes, and ROS1‐TKI treatment efficacy.

**Results:**

Compared to *TP53* non‐GOF mutations, patients with *TP53* GOF mutations showed higher mutation rates in *PIK3CA*, *STK11*, and *CTNNB1* but lower in *NTRK1*; increased *VEGFA* but decreased *DLL3* and *HRAS* amplification rates. Patients with *TP53* GOF mutations exhibited significantly higher tumor mutation burdens compared to those with non‐GOF *TP53* statuses. Patients with *TP53* mutations, both GOF and non‐GOF, showed significantly higher expression of immune checkpoints compared to *TP53* wild‐type patients. GOF‐mutated patients also had higher M1 macrophage and CD8+ T cell infiltration, along with elevated B cell receptor signaling. Consistent with our findings, analysis of external cohorts revealed that *TP53* GOF mutations were associated with improved prognosis in the context of immunotherapy. Among *ROS1* fusion‐positive patients treated with ROS1‐TKIs, those harboring *TP53* GOF mutations had a longer median PFS compared to patients with non‐GOF *TP53,* although both were shorter than those with wild‐type TP53. Additionally, *TP53* GOF mutations were associated with a relatively lower histologic grade than non‐GOF mutations.

**Conclusions:**

*TP53* GOF mutations were associated with poorer ROS1‐TKI treatment outcomes but improved immunotherapy response in NSCLC, with elevated immune activities and distinct molecular profiles.

**Abbreviations**
CI: confidence interval
CNV: copy‐number variation
FFPE: formalin‐fixed paraffin‐embedded
GOF: gain‐of‐function
GSEA: gene set enrichment analysis
HR: hazard ratio
INDEL: insertions/deletions
LOF: loss‐of‐function
MSI: microsatellite instability
NSCLC: non‐small cell lung cancer
OS: overall survival
PFS: progression‐free survival
SNV: single‐nucleotide variation
TKI: tyrosine kinase inhibitor
TMB: tumor mutational burden

## 1 Introduction

Lung cancer accounts for the most cancer‐related fatalities worldwide, with non‐small cell lung cancer (NSCLC) making up 80%–85% of all lung cancer cases [1]. Wild‐type p53 protein, which is encoded by the *TP53* gene, is a vital tumor suppressor involved in oncogenic stress response and tumor development inhibition by activating the transcription of genes driving signaling processes, including DNA repair, cell cycle, cell senescence, apoptosis, and cell metabolisms [2, 3, 4]. *TP53* gene mutations, with an overall frequency of 50%, are the most frequently identified genetic alteration across all cancer types and exhibit a diverse spectrum of alteration subtypes, such as deletions, missense, nonsense, and frameshifts [4, 5, 6]. *TP53* mutations are widely recognized as a negative prognostic indicator in various cancer types, while their predictive value across different treatments is highly diversified [7, 8, 9, 10, 11].

*TP53* mutations can be classified into three not mutually exclusive subtypes based on the functional impact of the p53 mutant proteins. Loss‐of‐function (LOF) mutations result in the loss of tumor‐suppressive activities normally regulated by wild‐type p53 proteins [4]. Dominant‐negative effects occur when mutant p53 proteins not only lose the tumor‐suppressive functions but also inhibit the tumor‐suppressive activity of the wild‐type p53 allele through hetero‐oligomerization [12]. Gain‐of‐function (GOF) activities result from neomorphic properties acquired by mutant p53 proteins, driving oncogenic processes such as promoting tumor progression and treatment resistance—functions not originally regulated by the wild‐type protein [13, 14, 15]. While multiple studies have confirmed the critical roles of LOF and dominant‐negative effect events in tumorigenesis and malignant transformation, the effects of GOF activities remain less well‐understood [16, 17, 18]. A previous study reported that tumor development, survival, and invasion characteristics are more closely associated with the LOF properties of *TP53* mutations than the GOF properties [19].

Despite ongoing debate, *TP53* GOF activities are clinically validated as being associated with cancer survival and treatment efficacy across various cancer types and therapies, with potential to serve as a therapeutic target. In patients with advanced pancreatic ductal adenocarcinoma, the presence of *TP53* GOF mutations was associated with worse overall survival (OS) compared to those with *TP53* non‐GOF mutations (hazard ratio [HR], 1.27; 95% confidence interval [CI], 1.02–1.59) and wild‐type *TP53* (HR, 1.24; 95% CI, 0.98–1.57) [20]. Interestingly, in metastatic colorectal cancer, *TP53* GOF mutations served as biomarkers for shorter OS in left‐sided colorectal cancer but were not prognostic in right‐sided colorectal cancer [21]. *TP53* GOF mutations can also contribute to resistance against osimertinib, a third‐generation EGFR tyrosine kinase inhibitor (TKI), by inducing TNF‐α expression [22]. Previous studies have shown that *TP53* R248Q mutations, a type of GOF mutation, produce mutant p53 proteins that bind to Stat3 and activate Jak2/Stat3 signaling, promoting tumor development and invasion, while ablation of the mutated allele can suppress such processes [23].

Despite significant progress in understanding the characteristics and functions of *TP53* GOF mutations, their clinical and molecular features, as well as their role in treatment efficacy and resistance in lung cancer, remain poorly understood. In this study, we comprehensively analyzed the molecular characteristics of *TP53* GOF mutations in our NSCLC cohort and assessed their impact on immunotherapy outcome, ROS1‐TKI treatment efficacy, and histologic grade through extensive analysis of multiple external cohorts.

## 2 Methods

### 2.1 Study Cohort Enrollment and Data Collection

Patients admitted at participating hospitals from January 2020 to March 2024 were retrospectively screened. The enrollment criteria were set as follows: (a) age 18 years or older; (b) confirmed histological diagnosis of NSCLC according to the 2016 World Health Organization Classification of Tumors; (c) available baseline tumor DNA sequencing data (GeneseeqPrime panel targeting 437 cancer‐related genes, Nanjing Geneseeq Technology Inc., Nanjing, China); (d) complete medical records. This study was approved by the Medical Ethics Committee of Nanjing Geneseeq Medical Laboratory (NSJB‐MEC‐2024‐14) and was conducted in accordance with the Declaration of Helsinki. Informed consent was obtained from all the participating patients.

### 2.2 DNA Extraction, Library Preparation and Targeted DNA Sequencing

Genomic DNA was extracted from formalin‐fixed paraffin‐embedded (FFPE) tumor samples using the QIAamp DNA FFPE Tissue Kit (Qiagen). Purified genomic DNA was qualified by Nanodrop 2000 spectrophotometer (Thermo Fisher Scientific) for A260/A280 and A260/A230 ratios and DNA quantities were assessed with Qubit 3.0 fluorometer (Thermo Fisher Scientific). Samples with A260/280 ratios between 1.8–2.0 and A260/A230 ratios above 1.8 were acceptable for library preparations. Genomic DNA from the white blood cells collected from the same patient was also analyzed as the normal control for germline variants and clonal hematopoiesis mutation filtering [24]. Sequencing libraries were prepared using the KAPA Hyper Prep Kit (KAPA Biosystems), and hybridization‐based target enrichment was performed with the GeneseeqPrime pan‐cancer gene panel and the xGen Lockdown Hybridization and Wash Reagents Kit (Integrated DNA Technologies). Library capture was completed using Dynabeads M‐270 (Life Technologies), followed by amplification with KAPA HiFi HotStart ReadyMix (KAPA Biosystems). Library quantification was done via qPCR and the KAPA Library Quantitative Kit (KAPA Biosystems). Library fragment size was determined by Bioanalyzer 2100 (Agilent Technologies). Finally, the libraries were sequenced on an Illumina HiSeq4000 platform (Illumina) according to the manufacturer's instructions.

### 2.3 Bioinformatics Analysis

Quality control of FASTQ files was performed using Trimmomatic [25], which removed leading or trailing low‐quality bases (with quality score below 20) and eliminated *N* bases. Sequencing data were then aligned to the human reference genome (hg19) using the Burrows‐Wheeler Aligner (BWA‐mem, v0.7.12) [26], and alignment results were deduplicated with Sambamba. Base quality recalibration and indel realignment were carried out using the Genome Analysis Toolkit (GATK 3.4.0) [27]. Variant calling for single‐nucleotide variations (SNVs) and insertions/deletions (INDELs) was performed with VarScan2 [28]. Genomic fusions were detected using FACTERA [29] with default settings, while copy‐number variations (CNVs) were identified with CNVkit [30] using default parameters, applying thresholds of 0.6 for copy‐number loss and 2.0 for copy‐number gain. Tumor mutational burden (TMB) was calculated as the number of somatic, coding base substitutions and indel mutations per megabase of genome analyzed [31]. Microsatellite instability (MSI) was defined as a sample exhibiting instability in more than 40% of 52 indel sites covered by the panel [32].

### 2.4 Definition of TP53 Gain‐of‐function Mutations

The *TP53* GOF mutations are defined according to the “The *TP53* Database” where gain‐of‐function activities were assessed in various experimental assays (https://tp53.cancer.gov/view_data?bq_view_name=FunctionDownload). Mutations annotated as having gain‐of‐function activities were classified as *TP53* GOF mutations in this study (full list of *TP53* variations in Supporting Information).

### 2.5 External Cohorts and Data Collection

**FIGURE 1:**
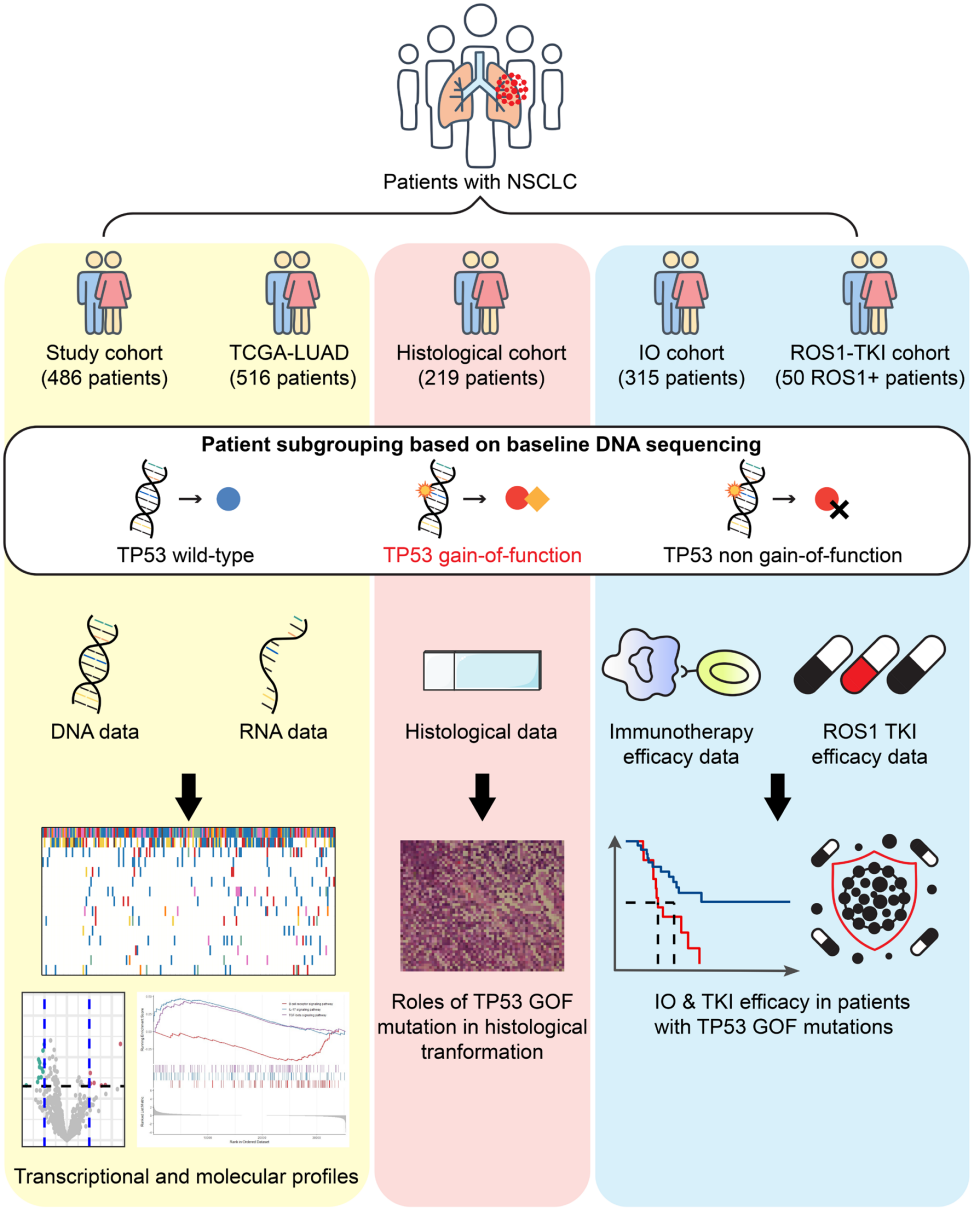
Workflow and enrolled cohorts of the study. Study design and cohort analysis including DNA and RNA sequencing data.

The DNA and bulk‐RNA sequencing data from 516 patients in the TCGA‐LUAD cohort (https://portal.gdc.cancer.gov/projects/tcga‐luad) were evaluated to explore the expression profiles of *TP53* GOF mutations. Clinical outcomes and data from external NSCLC cohorts treated with immunotherapy [33, 34], ROS1 tyrosine kinase inhibitors [35], and with histologic data [36] were analyzed to assess relationships between *TP53* GOF mutations and immunotherapy outcomes, ROS1‐TKI treatment efficacy, and histologic subtypes (Figure 1).

### 2.6 Statistical Analysis

Genomic and transcriptomic comparisons were conducted using Fisher's exact test to evaluate differences in categorical variables across groups and the Wilcoxon rank‐sum test to analyze differences in continuous variables. Differential gene expression analysis was performed using the “DESeq2” package in R, with transcripts classified as differentially expressed if they had an absolute log2 fold change ≥ 2 and an adjusted *p* value < 0.05. Gene set enrichment analysis (GSEA) was conducted with “clusterProfiler” package in R. The estimation of the proportions of tumor‐infiltrating immune cells was performed using xCell [37]. Progression‐free survival (PFS) and overall survival (OS) curves, along with 95% confidence intervals, were generated using the Kaplan–Meier method, with group comparisons performed via the log‐rank test. Hazard ratios were calculated using univariate Cox proportional hazards regression models. A two‐tailed *p* value < 0.05 was considered statistically significant unless otherwise stated. All statistical analyses were performed using *R* software (v4.4.2).

## 3 Results

### 3.1 Study Cohort and Patient Characteristics

The study enrolled 486 patients diagnosed with NSCLC from January 2020 to March 2024 with available clinical and baseline DNA sequencing data. The study cohort had a median age of 60 years, with 54.5% male patients. Most patients have lung adenocarcinoma (86.4%, 420/486) and stage IV disease (48.8%, 237/486). Among all patients, 98 were identified with *TP53* GOF mutations (20.2%), 189 with *TP53* non‐GOF mutations (38.9%), and 199 with *TP53* wild‐type (40.9%). There were no significant differences in sex or age distribution among patients with *TP53* GOF, non‐GOF, and wild‐type mutations, although those with wild‐type *TP53* had a higher proportion of stage I disease and lung adenocarcinoma histologic subtypes (Table S1).

### 3.2 Molecular Characteristics of NSCLC Patients With *TP53* GOF Mutations

**FIGURE 2:**
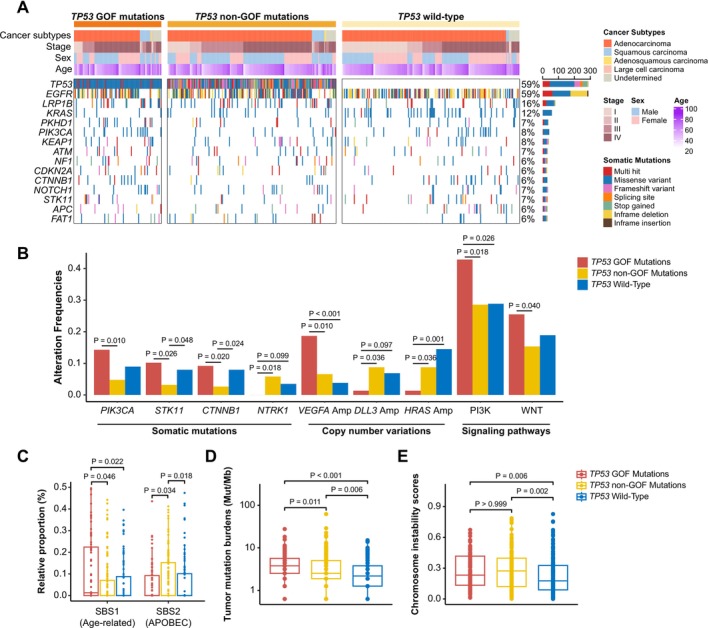
Molecular profiles of non‐small cell lung cancer (NSCLC) patients with *TP53* gain‐of‐function (GOF) mutations, non‐GOF mutations, and wild‐type *TP53*. (A) Oncoplot showing Molecular landscapes of NSCLC patients with *TP53* gain‐of‐function (GOF) mutations, non‐GOF mutations, and wild‐type *TP53*. (B) Bar plot showing differences in mutation, copy number variation, and signaling pathway alteration frequencies among patients with *TP53* gain‐of‐function (GOF) mutations, non‐GOF mutations, and wild‐type *TP53*. (C–E) Box plot showing differences in (C) mutational signatures, (D) tumor mutation burdens, and (E) chromosomal instability scores among patients with *TP53* gain‐of‐function (GOF) mutations, non‐GOF mutations, and wild‐type *TP53*.

The mutational profiles of patients with different *TP53* mutated features are shown in Figure 2A. The most mutated genes in the study cohorts were *TP53* (59%), *EGFR* (59%), *LRP1B* (16%), *KRAS* (12%), and *PKHD1* (7%). Patients with *TP53* GOF mutations, compared to those with *TP53* non‐GOF mutations, exhibited a higher frequency of *PIK3CA* (*p* = 0.010), *STK11* (*p* = 0.026), and *CTNNB1* (*p* = 0.020) mutations, *VEGFA* copy number amplifications (*p* = 0.010), and alterations in the PI3K (*p* = 0.018) and WNT (*p* = 0.040) signaling pathways, while showing lower frequencies of *NTRK1* mutations (*p* = 0.018), *DLL3* (*p* = 0.036) and *HRAS* amplifications (*p* = 0.036) (Figure 2B). Age‐related mutational signatures (SBS1) were enriched in patients with *TP53* GOF mutations (*p* = 0.046), whereas APOBEC‐related signatures (SBS2) were more prominent in patients with non‐GOF *TP53* mutations (*p* = 0.034, Figure 2C). Interestingly, patients with *TP53* GOF mutations exhibited the highest TMB, compared to those with non‐GOF mutations (*p* = 0.011), while *TP53* wild‐type patients had the lowest (*p* = 0.006, Figure 2D). Moreover, patients with *TP53* GOF and non‐GOF mutations had similar chromosomal instability scores (*p* > 0.999), both of which were higher than those observed in patients with *TP53* wild‐type (*p* = 0.006 and 0.002, respectively; Figure 2E).

### 3.3 The Actionable Mutation Profiles of Patients With *TP53* GOF Mutations

**FIGURE 3:**
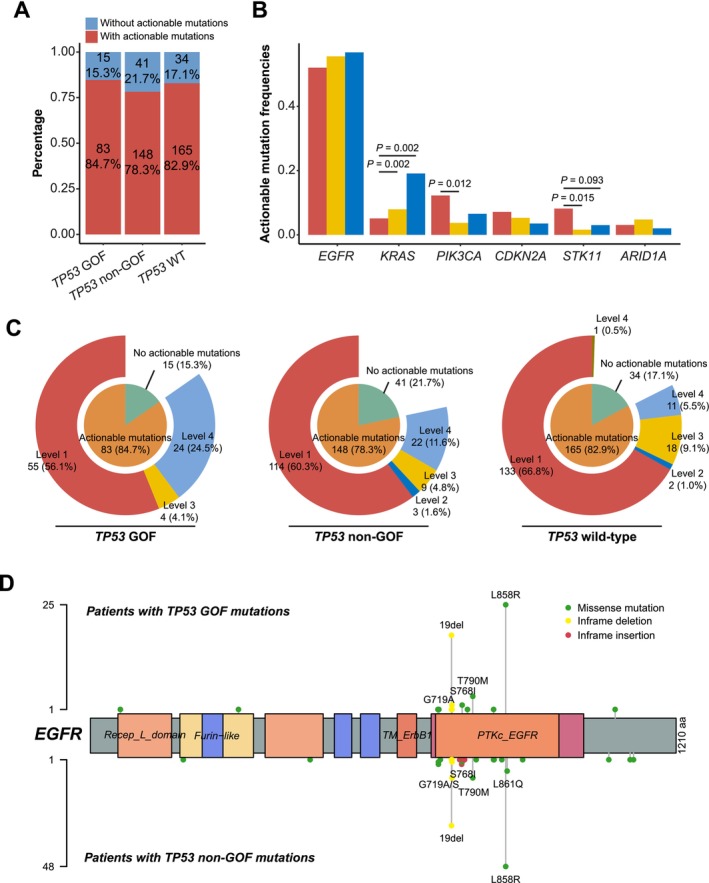
The differences in actionable mutation profiles among non‐small cell lung cancer (NSCLC) patients with *TP53* gain‐of‐function (GOF) mutations, non‐GOF mutations, and wild‐type *TP53*. (A) Stacked bar plot showing percentages of patients carrying actionable mutations; (B) Bar plot showing differences in actionable mutation frequencies among NSCLC patients with *TP53* GOF mutations, non‐GOF mutations, and wild‐type *TP53*; (C) Pie charts showing distributions of actionable mutation levels in NSCLC patients with *TP53* GOF mutations, non‐GOF mutations, and wild‐type *TP53*; (D) Lollipop plot showing the distribution of *EGFR* mutation loci in NSCLC patients with *TP53* GOF mutations and non‐GOF mutations.

We further analyzed the actionable mutation profiles of patients with *TP53* GOF mutations in our study cohort. Levels of actionability were categorized based on accessibility to the therapeutic levels defined in the OncoKB database (https://www.oncokb.org/), which reflect the accessibility and clinic relevance of targeted therapies. Level 1 mutations are linked to FDA‐approved drugs, while Level 4 mutations have limited supporting biological evidence. The proportion of patients with actionable mutations was comparable among those with *TP53* GOF mutations (84.7%, 83/98), non‐GOF mutations (78.3%, 148/189), and wild‐type *TP53* (82.9%, 165/199) (Figure 3A). Among the most common actionable mutations detected, patients with *TP53* GOF mutations showed fewer *KRAS* mutations (*KRAS* G12C, *p* = 0.002) but a higher prevalence of *STK11* mutations (*STK11* oncogenic mutations, *p* = 0.015) than other patients (Figure 3B). While the majority of patients carried Level 1 actionable mutations (56.1% in *TP53* GOF, 60.3% in *TP53* non‐GOF, and 66.8% in *TP53* wild‐type), patients with *TP53* GOF mutations exhibited a higher proportion of level 4 actionable mutations (24.5% in *TP53* GOF, 11.6% in *TP53* non‐GOF, and 5.5% in *TP53* wild‐type; Figure 3C). The *EGFR* mutational landscapes of patients at baseline with *TP53* GOF and non‐GOF mutations were similar, with L858R, exon 19 deletion, and T790M being the most common alterations (Figure 3D).

### 3.4 RNA Analyses Revealed That Patients With *TP53* GOF Mutations Had Higher Immune‐Related Activities

**FIGURE 4:**
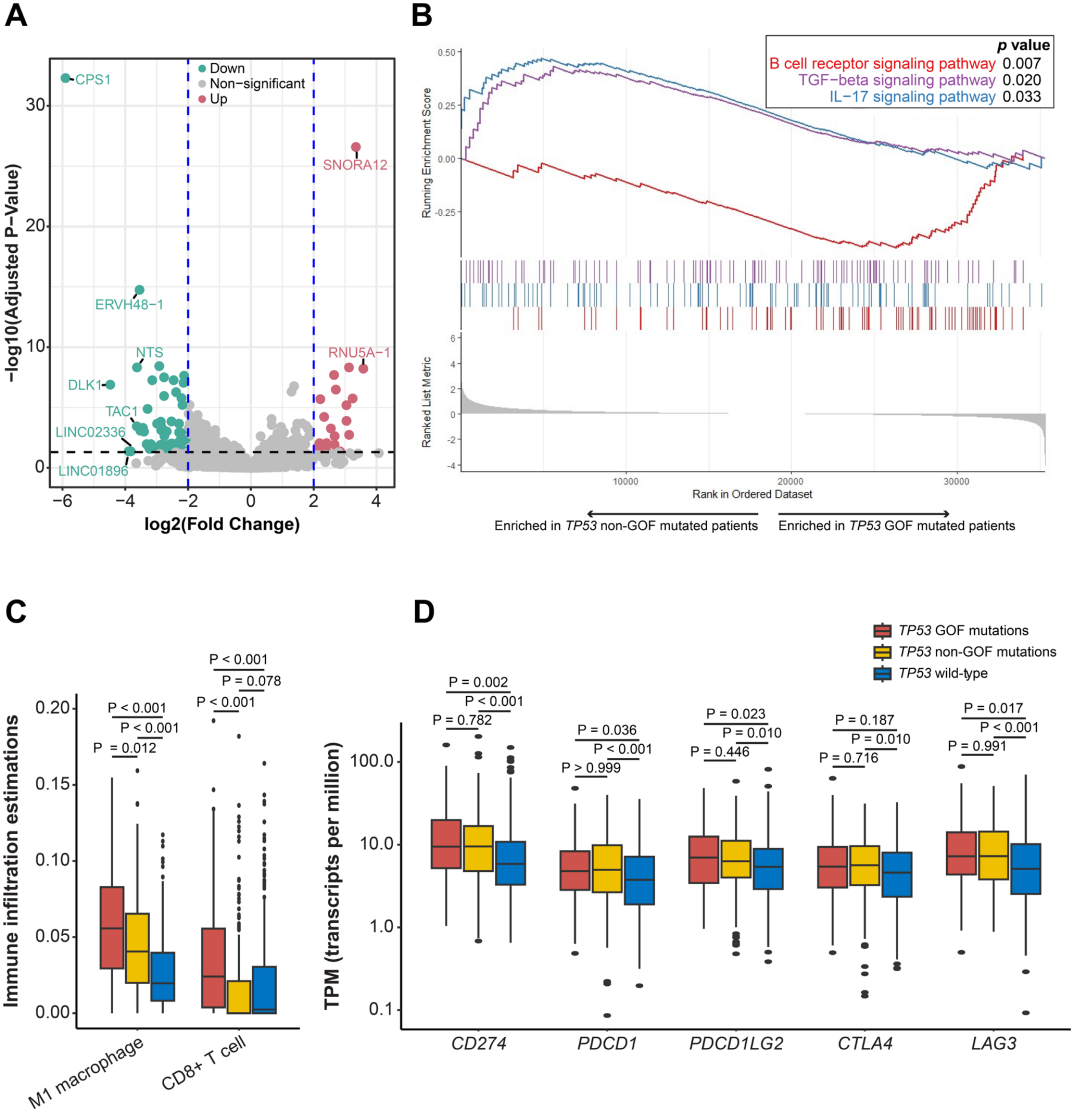
The differences in RNA expression profiles among non‐small cell lung cancer (NSCLC) patients with *TP53* gain‐of‐function (GOF) mutations, non‐GOF mutations, and wild‐type *TP53*. (A) Volcano plot showing up‐ and down‐regulated genes in NSCLC patients with *TP53* GOF mutations compared to those with non‐GOF mutations. (B) Gene‐set enrichment plot showing the enrichment of signaling pathway expressions in NSCLC patients with *TP53* GOF mutations and non‐GOF mutations. (C, D) Box plot showing the differences in (C) M1 macrophage and CD8 + T cell infiltration scores, as well as in (D) in immune checkpoint expressions in NSCLC patients with *TP53* GOF mutations, non‐GOF mutations, and wild‐type *TP53*.

To uncover the expression profiles and explore the potential tumor immune microenvironment alteration for patients with *TP53* GOF mutations, baseline genomic and transcriptomic sequencing data from the TCGA‐LUAD cohort with 516 patients were analyzed. Compared to patients with *TP53* non‐GOF mutations, those with *TP53* GOF mutations showed significant upregulation of *SNORA12* and *RNU5A‐1*, and downregulation of *CPS1*, *ERVH48‐1*, *NTS*, *DLK1*, and *TAC1* (Figure 4A). The full list of up‐ and down‐regulated genes is given in Table S2. In the GSEA analysis, patients with *TP53* GOF mutations showed elevated B cell receptor signaling pathway (*p* = 0.007) but lower TGF‐beta (*p* = 0.020) and IL‐17 (*p* = 0.033) signaling pathways (Figure 4B). Notably, in tumor immune infiltration analyses, patients with TP53 gain‐of‐function mutations exhibited significantly higher M1 macrophages and CD8+ T cell infiltration (Figure 4C). The expression levels of common immune checkpoints, including *CD274*, *PDCD1*, *PDCD1LG2*, *CTLA4*, and *LAG3*, were comparable among *TP53*‐mutated patients but significantly higher than those with wild‐type (Figure 4D).

### 3.5 Associations of *TP53* GOF Mutations With Histologic Subtypes and Survival Outcomes Across Different Treatments

**FIGURE 5:**
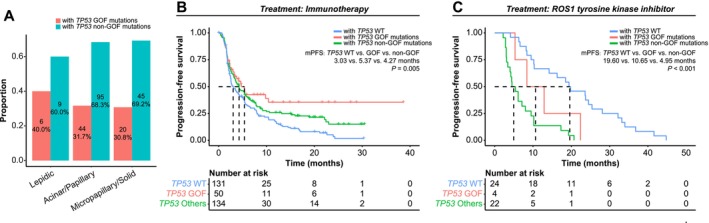
Effects of *TP53* mutation subtypes on survival outcomes and histologic grade in non‐small cell lung cancer (NSCLC) patients. (A) Stacked bar plot showing the percentages of patients with TP53 gain‐of‐function (GOF) and non‐GOF mutations across lepidic, acinar/papillary, and solid/micropapillary histologic subtypes. (B) Kaplan–Meier estimates of progression‐free survival of NSCLC patients with *TP53* GOF mutations, non‐GOF mutations, and wild‐type *TP53* treated with immunotherapy. (C) Kaplan–Meier estimates of progression‐free survival of ROS1 fusion‐positive NSCLC patients with *TP53* GOF mutations, non‐GOF mutations, and wild‐type *TP53* treated with ROS1 tyrosine kinase inhibitors.

We aim to investigate the roles of *TP53* GOF mutations in different histologic subtypes using data from a 219 patients' cohort with detailed histologic profiles [36]. In the histological analysis, patients were classified into low‐ (lepidic), intermediate‐ (acinar/papillary), and high‐grade (solid/micropapillary) histologic subtypes [36]. Higher‐grade tumors are characterized by a higher rate of genomic alteration and greater chromosomal instability [36]. In patients with *TP5*3 mutations, the proportion of those carrying GOF mutations decreased with increasing histologic grades (40.0% in lepidic, 31.7% in acinar/papillary, and 30.8% in solid/micropapillary; Figure 5A). To assess the impact of *TP53* GOF mutations on treatment outcomes, detailed immunotherapy response data from two combined cohorts comprising 315 patients—one cohort treated with anti‐PD1 or anti‐PDL1 therapy [34] and the other with a combination of PD‐1 and CTLA‐4 blockade [33] were analyzed. In addition, a cohort of 50 *ROS1* fusion‐positive patients treated with first‐line ROS1‐TKIs (crizotinib and lorlatinib) was evaluated [35]. Tyrosine kinase inhibitors (TKIs) are widely used as first‐line targeted therapies for cancers driven by actionable tyrosine kinase alterations. Inhibitors targeting kinases such as EGFR, ALK, ROS1, PDGF, and VEGFR have contributed significantly to advances in cancer treatment. Among these, NSCLC patients with *ROS1* gene fusions represent a subpopulation highly sensitive to TKI treatment [38]. Patients with *TP53* mutations had prolonged PFS under immunotherapy compared to *TP53* wild‐type patients, with those carrying GOF mutations exhibiting the longest median PFS (mPFS: 3.03 months for wild‐type, 4.27 months for non‐GOF mutations, and 5.37 months for GOF mutations; Figure 5B), while none of the clinical factors, except TMB, were associated with the PFS of immunotherapy (Table S3). When adjusting for TMB, patients with *TP53* GOF mutations remained having superior PFS compared to those with wild‐type counterparts (HR, 0.63; 95% CI, 0.41–0.99; *p* = 0.043), whereas the difference in comparison to those with non‐GOF mutations was not statistically significant (HR, 0.78; 95% CI, 0.52–1.18; *p* = 0.246). For patients treated with ROS1‐TKIs, *TP53* mutations were a negative biomarker for PFS, although those with GOF mutations had longer median PFS than those with non‐GOF mutations (mPFS: 19.60 months for wild‐type, 4.95 months for non‐GOF mutations, and 10.65 months for GOF mutations; Figure 5C).

## 4 Discussion

Previous studies have demonstrated that mutant p53 with GOF properties can promote tumor cell proliferation, survival, metastasis, and resistance to cancer therapies [39]. The functional alterations of GOF‐p53 mutant proteins are diverse and complex, driving oncogenicity by synergizing with other oncogenic mutations or activating multiple cancer‐related pathways [40, 41]. *TP53* GOF mutations have been identified as prognostic factors in highly methylated metastatic colorectal cancer, advanced pancreatic ductal adenocarcinoma, and left‐sided metastatic colorectal cancer with varying clinical outcomes [20, 21, 42], while their roles in NSCLC patients remain unclear. We comprehensively analyzed the molecular and expression profiles of NSCLC patients with *TP53* GOF mutations and explored their roles in histologic pattern change and treatment efficacy.

Regarding the molecular profiles, compared to those with non‐GOF mutations, patients with *TP53* GOF mutations exhibited higher frequencies of *PIK3CA*, *STK11*, and *CTNNB1* mutations, *VEGFA* copy number amplifications, and alterations in the PI3K and WNT signaling pathways, while showing lower frequencies of *NTRK1* mutations, *DLL3*, and *HRAS* amplifications. *PIK3CA*, *STK11*, and *CTNNB1*, genes commonly mutated in lung cancer, have been positively linked to the activation of key oncogenic pathways—including the cell cycle, RTK/RAS, PI3K, P53, and WNT—which drive tumor proliferation. These mutations have also been linked to poorer prognosis and resistance to immunotherapy and EGFR‐TKIs [43, 44, 45, 46]. Although these gene mutations are generally associated with tumor progression, their effects on immunotherapy response remain under debate. *PIK3CA* mutations have been shown to increase *PD‐L1* expression and TMB, potentially increasing sensitivity to immune checkpoint inhibitors [47, 48, 49, 50]. A study has suggested better survival outcomes in patients harboring *STK11* after immunotherapy treatment [51]. In our analysis, *TP53* GOF mutations were significantly associated with increased mutation rates in several of these genes (*PIK3CA, STK11 and CTNNB1*), while patients with *TP53* GOF mutations demonstrated improved responses to ICIs. This seemingly paradoxical observation may reflect a complex interplay of multiple molecular factors within the tumor microenvironment. *VEGFA* copy number amplifications, which are also enriched in *TP53* GOF‐mutated patients, have been shown to activate the PI3K/AKT, RAS/ERK, and STAT3 signaling pathways, thereby promoting tumor progression [52]. Genetic alterations enriched in *TP53* non‐GOF patients are rare in NSCLC, and their impact on prognosis and treatment efficacy remains poorly understood [53, 54]. In regard to actionable mutations, *TP53* GOF‐mutated patients exhibited significantly fewer *KRAS* (including *KRAS* G12C) mutations but a higher prevalence of *PIK3CA* and *STK11* mutations. Interestingly, studies have shown that *KRAS* G12C and *TP53* mutations predict better prognosis with immunotherapy in lung cancer patients [55, 56], while those with *TP53* GOF mutations are more likely to benefit from therapies targeting *PIK3CA* and *STK11*.

Expression analyses of the TCGA‐LUAD cohorts revealed that *CPS1* and *NTS*, oncogenic factors strongly associated with cancer neuroendocrine differentiation and tumor invasiveness, were significantly downregulated in *TP53* GOF mutated patients. Although CPS1 and NTS expression levels are typically positively correlated [57], our RNA analysis indicates these oncogenic processes are suppressed in lung cancer patients harboring *TP53* GOF mutations. GSEA and tumor infiltration xCell analyses revealed that patients with *TP53* GOF mutations exhibit significantly higher expression of the B cell receptor signaling pathway, increased infiltration of CD8+ T cells and macrophages, along with higher expression of immune checkpoint genes, indicating a hotter immune microenvironment. Analysis of external NSCLC cohorts indicated that patients with *TP53* GOF mutations experienced prolonged PFS with immunotherapy treatments. Previous research found that *TP53* GOF‐mutated tumors are enriched with CD8+ and CD4+ T cells exhibiting high PD‐1 expression, which shows responsiveness to immune checkpoint inhibitors, highlighting the potential of immunotherapy in *TP53* GOF‐mutated patients [58].

The analysis of the histological cohort showed a decline in the percentage of patients with *TP53* GOF mutations within the *TP53*‐mutated group as the histologic grade increased. Previous studies revealed that tumors with *TP53* GOF mutations exhibited loss of apical‐basal polarity and glandular structure at protrusion sites [59], although their role in histologic changes remains unclear. While previous literature validated that *TP53* GOF mutations can promote resistance to EGFR‐TKIs [22], and *TP53* mutations were associated with shorter PFS for patients treated with ROS1 and EGFR‐TKIs [35, 60], our findings reveal that patients with *TP53* GOF mutations had longer PFS than those with non‐GOF *TP53* mutations but shorter PFS than patients with wild‐type *TP53*.

Despite the strengths of our study in leveraging multiple datasets to characterize the impact of *TP53* GOF mutations on genetic alterations, histologic features, and therapeutic responses, several limitations must be acknowledged. First, the lack of clinical treatment data within our enrolled study cohort restricts our ability to directly correlate molecular alterations with the clinical outcomes in the study cohort. Second, due to the limited number of patients harboring *TP53* GOF mutations in the cohort, we could not evaluate the impact of *TP53* GOF mutations on immunotherapy response in PD‐L1 high patients. It is important to note that the predictive value of individual gene mutations remains limited. Immunotherapy response is determined by multiple factors. Immunotherapy response is likely driven by a combination of genomic, transcriptomic, and microenvironmental features. Therefore, to better predict ICIs, beyond molecular alterations, TMB, expression levels of immune checkpoint proteins, and other factors should be considered. Although our analysis indicates that patients with *TP53* GOF mutations showed increased CD8+ T cell infiltration, higher TMB, and better response to ICIs, these findings are insufficient to determine whether *TP53* GOF mutations can serve as a reliable predictive biomarker for treatment efficacy. Further prospective studies in larger cohorts and functional validations are required for use in clinical settings.

## 5 Conclusions

In summary, this study comprehensively analyzed the molecular profiles and treatment outcomes of NSCLC patients with *TP53* GOF mutations, revealing that these mutations are associated with improved survival compared to non‐GOF *TP53* mutations when treated with immunotherapy or ROS1‐TKIs, while the percentage of patients harboring *TP53* GOF mutations was lower in patients with a higher grade of histologic subtype.

## Author Contributions

**Zheng Zhao:** formal analysis, software, investigation, writing – original draft, writing – review and editing, visualization. **Rui Lu:** writing – original draft, visualization, writing – review and editing, software, formal analysis. **Feng Zhang:** formal analysis, investigation, writing – review and editing, visualization. **Hanlin Chen:** formal analysis, visualization, writing – review and editing. **Qiuxiang Ou:** methodology, writing – review and editing. **Xiaotian Zhao:** writing – review and editing. **Xinyue Hong:** writing – review and editing. **Hua Bao:** writing – review and editing, methodology. **Degan Lu:** conceptualization, methodology, validation, resources, project administration, writing – review and editing, supervision. **Jie Min:** conceptualization, methodology, validation, resources, project administration, writing – review and editing, supervision.

## Ethics Statement

This study was approved by the Medical Ethics Committee of Nanjing Geneseeq Medical Laboratory (NSJB‐MEC‐2024‐14) and written informed consents from all patients were collected. The work was carried out in accordance with The Declaration of Helsinki.

## Consent

The authors have nothing to report.

## Conflicts of Interest

Hanlin Chen, Qiuxiang Ou, Xiaotian Zhao, Xinyue Hong, and Hua Bao are employees of Nanjing Geneseeq Technology Inc. The authors declare no conflicts of interest.

## Supporting information


**Table S1:** Clinical characteristics of the study cohort.
**Table S2:** Differential expressed genes in patients with TP53 gain‐of‐function compared to those with TP53 other mutations in the TCGA‐LUAD cohort.
**Table S3:** Univariate and multivariate analyses of progression‐free survival for the immunotherapy cohort.

**Data S1:** Supporting Information.

## Data Availability

The data that support the findings of this study are available from the corresponding author upon reasonable request.
