# Supplementary material for: Molecular Characteristics and Treatment Implications of TP53 Gain‐of‐Function Mutations in Non‐Small Cell Lung Cancer
Source: Cancer Med. 2025 Oct 23;14(20):e71215. doi: 10.1002/cam4.71215 (PMC12547478; doi:10.1002/cam4.71215)
Supplement: Supplementary file 1 — Table S1: Clinical characteristics of the study cohort. Table S2: Differential expressed genes in patients with TP53 gain‐of‐function compared to those with TP53 other mutations in the TCGA‐LUAD cohort. Table S3: Univariate and multivariate analyses of progression‐free survival for the immunotherapy cohort. [file CAM4-14-e71215-s001.docx]

**Supplementary Information**

**Table S1: Clinical characteristics of the study cohort.**

|  | **With *TP53* GOF mutations**  **(N = 98)** | **With *TP53* non-GOF mutations**  **(N = 189)** | **With *TP53* WT**  **(N = 199)** | ***P*** |
| --- | --- | --- | --- | --- |
| **Age** |  |  |  | 0.817 |
| ≤60 years | 47 | 101 | 99 |  |
| >60 years | 51 | 88 | 100 |  |
| Median (range) | 61 (32-86) | 60 (27-89) | 61 (26-84) |  |
|  |  |  |  |  |
| **Sex** |  |  |  | 0.217 |
| Male | 55 | 117 | 106 |  |
| Female | 43 | 72 | 93 |  |
|  |  |  |  |  |
| **Stage** |  |  |  | <0.001 |
| I | 12 | 26 | 76 |  |
| II | 3 | 16 | 17 |  |
| III | 20 | 55 | 24 |  |
| IV | 63 | 92 | 82 |  |
|  |  |  |  |  |
| **Histological subtypes** |  |  |  | <0.001 |
| Adenocarcinoma | 74 | 162 | 184 |  |
| Squamous carcinoma | 12 | 14 | 3 |  |
| Adenosquamous carcinoma | 1 | 2 | 0 |  |
| Large cell carcinoma | 1 | 0 | 0 |  |
| Unknown | 10 | 11 | 12 |  |

Note: GOF, gain-of-function; WT, wild-type.

**Table S2: Differential expressed genes in patients with TP53 gain-of-function compared to those with TP53 other mutations in the TCGA-LUAD cohort.**

| **Gene** | **Base Mean** | **log2(Fold Change)** | ***P*** | ***P* adjusted** | **Type** |
| --- | --- | --- | --- | --- | --- |
| *CPS1* | 1.02e4 | -5.90 | 1.32e-37 | 4.99e-33 | Down |
| *SNORA12* | 8.17 | 3.34 | 1.36e-31 | 2.56e-27 | Up |
| *ERVH48-1* | 106.46 | -3.54 | 1.42e-19 | 1.79e-15 | Down |
| *COL25A1* | 183.20 | -2.92 | 3.96e-13 | 3.74e-09 | Down |
| *NTS* | 457.74 | -3.63 | 6.36e-13 | 4.79e-09 | Down |
| *SNORA74A* | 2.45 | 3.12 | 7.61e-13 | 4.79e-09 | Up |
| *RNU5A-1* | 2.63 | 3.58 | 1.13e-12 | 6.12e-09 | Up |
| *RN7SL546P* | 1.99 | 2.65 | 4.30e-12 | 2.03e-08 | Up |
| *VTN* | 79.68 | -2.13 | 5.55e-12 | 2.33e-08 | Down |
| *MTND1P23* | 325.25 | -2.77 | 8.39e-12 | 3.17e-08 | Down |
| *FGA* | 1.03e4 | -3.14 | 1.65e-11 | 5.52e-08 | Down |
| *KRT81* | 489.76 | -2.46 | 1.75e-11 | 5.52e-08 | Down |
| *TRIM72* | 34.53 | -2.14 | 3.10e-11 | 9.01e-08 | Down |
| *DLK1* | 78.19 | -4.48 | 4.81e-11 | 1.30e-07 | Down |
| *RN7SL664P* | 1.52 | 2.71 | 1.39e-10 | 3.29e-07 | Up |
| *PCSK1* | 384.51 | -2.38 | 2.56e-10 | 5.37e-07 | Down |
| *PAEP* | 2.21e3 | -2.76 | 5.62e-10 | 1.12e-06 | Down |
| *AC087273.2* | 34.26 | -2.21 | 8.95e-10 | 1.69e-06 | Down |
| *MUC17* | 2.64 | 3.24 | 9.95e-10 | 1.79e-06 | Up |
| *REG4* | 18.92 | 2.21 | 1.19e-09 | 2.05e-06 | Up |
| *MAPK4* | 145.65 | -2.18 | 3.94e-09 | 6.20e-06 | Down |
| *RN7SL508P* | 0.98 | 3.04 | 4.28e-09 | 6.46e-06 | Up |
| *PRSS29P* | 11.54 | -3.29 | 9.1e-09 | 1.32e-05 | Down |
| *RN7SL836P* | 1.24 | 2.33 | 4.64e-08 | 6.05e-05 | Up |
| *OR6T1* | 0.86 | 3.05 | 1.10e-07 | 1.29e-04 | Up |
| *F2* | 25.80 | -2.53 | 1.26e-07 | 1.44e-04 | Down |
| *KLK12* | 335.38 | -2.87 | 1.36e-07 | 1.51e-04 | Down |
| *TKTL1* | 45.37 | -2.29 | 2.39e-07 | 2.20e-04 | Down |
| *CPLX2* | 407.22 | -2.99 | 2.39e-07 | 2.20e-04 | Down |
| *TAC1* | 30.31 | -3.63 | 4.47e-07 | 3.67e-04 | Down |
| *LINC02617* | 7.65 | -2.84 | 6.34e-07 | 4.67e-04 | Down |
| *INSL4* | 80.95 | -3.44 | 6.90e-07 | 4.92e-04 | Down |
| *ADGRG7* | 10.41 | 2.55 | 7.98e-07 | 5.48e-04 | Up |
| *AC068643.1* | 7.74 | -2.83 | 9.84e-07 | 6.41e-04 | Down |
| *CNMD* | 104.91 | -2.86 | 1.22e-06 | 7.53e-04 | Down |
| *NEUROD1* | 28.36 | -3.42 | 1.44e-06 | 8.41e-04 | Down |
| *LINC02418* | 111.00 | -3.52 | 1.45e-06 | 8.41e-04 | Down |
| *LIN28A* | 20.82 | -2.68 | 1.69e-06 | 9.69e-04 | Down |
| *S100A7A* | 4.93 | -3.40 | 1.81e-06 | 0.001 | Down |
| *ZMAT4* | 51.17 | -2.29 | 2.23e-06 | 0.001 | Down |
| *PRR20G* | 7.17 | -2.22 | 2.45e-06 | 0.001 | Down |
| *UGT1A10* | 18.92 | 3.13 | 3.85e-06 | 0.002 | Up |
| *CALML5* | 96.86 | -2.76 | 4.81e-06 | 0.002 | Down |
| *GSTA7P* | 2.37 | 2.67 | 5.38e-06 | 0.002 | Up |
| *ATP11AUN* | 7.74 | -2.05 | 6.05e-06 | 0.003 | Down |
| *UNC5D* | 19.11 | -2.12 | 1.41e-05 | 0.005 | Down |
| *SP3P* | 56.14 | -2.10 | 1.52e-05 | 0.006 | Down |
| *SLC30A10* | 11.13 | -2.27 | 1.74e-05 | 0.006 | Down |
| *BX276092.9* | 5.53 | -2.22 | 2.99e-05 | 0.008 | Down |
| *DGKK* | 3.92 | -2.04 | 3.12e-05 | 0.009 | Down |
| *RN7SL116P* | 2.08 | 2.41 | 3.52e-05 | 0.009 | Up |
| *Metazoa_SRP* | 0.83 | 2.18 | 3.72e-05 | 0.009 | Up |
| *GPR26* | 5.35 | -2.29 | 4.10e-05 | 0.010 | Down |
| *AL024497.1* | 2.87 | -2.52 | 4.08e-05 | 0.010 | Down |
| *ETNPPL* | 33.90 | -2.81 | 4.80e-05 | 0.011 | Down |
| *KCNU1* | 11.38 | -3.18 | 4.75e-05 | 0.011 | Down |
| *SOX14* | 10.59 | -3.32 | 4.93e-05 | 0.011 | Down |
| *GP2* | 38.44 | -2.58 | 5.08e-05 | 0.011 | Down |
| *LCN15* | 14.68 | -2.44 | 5.10e-05 | 0.011 | Down |
| *AP000802.1* | 2.57 | -2.09 | 5.30e-05 | 0.011 | Down |
| *RN7SL555P* | 0.56 | 2.66 | 5.60e-05 | 0.012 | Up |
| *CHAT* | 26.03 | -2.10 | 5.79e-05 | 0.012 | Down |
| *MAGEA1* | 106.79 | -2.88 | 6.17e-05 | 0.013 | Down |
| *AC074389.2* | 3.07 | -2.86 | 1.06e-04 | 0.019 | Down |
| *SNORA74D* | 1.40 | 2.12 | 1.07e-04 | 0.019 | Up |
| *AL645608.3* | 2.92 | -2.29 | 1.22e-04 | 0.021 | Down |
| *PNMA6F* | 1.91 | -3.24 | 1.27e-04 | 0.021 | Down |
| *AC023824.6* | 4.96 | -3.07 | 1.32e-04 | 0.022 | Down |
| *AC007785.1* | 1.25 | -2.00 | 1.46e-04 | 0.023 | Down |
| *AC106799.3* | 13.97 | -2.33 | 1.70e-04 | 0.026 | Down |
| *MARCHF11* | 4.35 | -3.23 | 1.82e-04 | 0.027 | Down |
| *AC109635.6* | 3.01 | 2.39 | 1.95e-04 | 0.029 | Up |
| *AP000345.3* | 1.78 | -2.84 | 2.12e-04 | 0.030 | Down |
| *SSX1* | 16.83 | -2.83 | 2.96e-04 | 0.038 | Down |
| *AC016687.3* | 4.10 | 2.72 | 3.33e-04 | 0.041 | Up |
| *PAQR9* | 5.47 | -2.10 | 3.51e-04 | 0.043 | Down |
| *LINC01896* | 2.67 | -3.89 | 3.51e-04 | 0.043 | Down |
| *LINC02336* | 3.30 | -3.82 | 3.54e-04 | 0.043 | Down |
| *RN7SL735P* | 1.40 | 2.84 | 3.62e-04 | 0.044 | Up |
| *BX276092.7* | 4.42 | -2.18 | 4.10e-04 | 0.047 | Down |
| *NKX2-4* | 6.57 | -2.42 | 4.19e-04 | 0.047 | Down |
| *NDST4* | 2.70 | -2.24 | 4.16e-04 | 0.047 | Down |
| *AC104009.1* | 1.41 | -2.63 | 4.38e-04 | 0.048 | Down |
| *LINC01785* | 1.74 | -2.86 | 4.56e-04 | 0.050 | Down |

**Table S3: Univariate and multivariate analyses of progression-free survival for the immunotherapy cohort.**

| **Characteristics** | HR (95% CI) | | *P* |
| --- | --- | --- | --- |
| **Age (continuous)** | 1.00 | (0.99-1.00) | 0.584 |
| **Sex: female vs. male** | 1.08 | (0.84-1.38) | 0.559 |
| **Smoking status** |  |  |  |
| Current | Ref | -- | -- |
| Ever | 1.29 | (0.57-2.93) | 0.537 |
| Former | 0.67 | (0.28-1.61) | 0.372 |
| Never | 1.59 | (0.68-3.71) | 0.279 |
| **Histological subtypes** |  |  |  |
| Large cell carcinoma | Ref | -- | -- |
| Adenocarcinoma | 0.77 | (0.31-1.87) | 0.557 |
| Squamous cell carcinoma | 0.77 | (0.30-1.96) | 0.582 |
| **TMB:** ≥**10 vs. <10 muts/Mb** | 0.66 | (0.50-0.87) | **0.003** |

Note: TMB, tumor mutation burden; HR, hazard ratio; CI, confidence interval; Ref, reference.
