# Supplementary material for: Molecular Characteristics and Treatment Implications of TP53 Gain‐of‐Function Mutations in Non‐Small Cell Lung Cancer
Source: Cancer Med. 2025 Oct 23;14(20):e71215. doi: 10.1002/cam4.71215 (PMC12547478; doi:10.1002/cam4.71215)
Supplement: Supplementary file 2 — Data S1: Supporting Information. [file CAM4-14-e71215-s002.docx]

# Supplementary Data 1:

# TP53 Mutations List

| X | AAChange_Annotated | TP53_Status | Num_Patients | Mean_VAF |
| --- | --- | --- | --- | --- |
| 1 | p.R248W | TP53_GOF_Mutations | 8 | 0.210 |
| 2 | p.R273L | TP53_GOF_Mutations | 7 | 0.191 |
| 3 | p.R175H | TP53_GOF_Mutations | 6 | 0.252 |
| 4 | p.R248Q | TP53_GOF_Mutations | 6 | 0.601 |
| 5 | p.C176F | TP53_GOF_Mutations | 4 | 0.318 |
| 6 | p.R273C | TP53_GOF_Mutations | 4 | 0.227 |
| 7 | p.R273H | TP53_GOF_Mutations | 4 | 0.271 |
| 8 | p.R282W | TP53_GOF_Mutations | 4 | 0.259 |
| 9 | p.S241F | TP53_GOF_Mutations | 4 | 0.118 |
| 10 | p.Y220C | TP53_GOF_Mutations | 4 | 0.523 |
| 11 | p.G245D | TP53_GOF_Mutations | 3 | 0.335 |
| 12 | p.G245S | TP53_GOF_Mutations | 3 | 0.118 |
| 13 | p.C238Y | TP53_GOF_Mutations | 2 | 0.159 |
| 14 | p.H179R | TP53_GOF_Mutations | 2 | 0.096 |
| 15 | p.H179Y | TP53_GOF_Mutations | 2 | 0.091 |
| 16 | p.M237I | TP53_GOF_Mutations | 2 | 0.268 |
| 17 | p.R158H | TP53_GOF_Mutations | 2 | 0.202 |
| 18 | p.R249S | TP53_GOF_Mutations | 2 | 0.347 |
| 19 | p.V157F | TP53_GOF_Mutations | 2 | 0.298 |
| 20 | p.Y163C | TP53_GOF_Mutations | 2 | 0.167 |
| 21 | p.A138V | TP53_GOF_Mutations | 1 | 0.090 |
| 22 | p.C275F | TP53_GOF_Mutations | 1 | 0.021 |
| 23 | p.D281E | TP53_GOF_Mutations | 1 | 0.466 |
| 24 | p.E204* | TP53_GOF_Mutations | 1 | 0.092 |
| 25 | p.E224* | TP53_GOF_Mutations | 1 | 0.075 |
| 26 | p.E258K | TP53_GOF_Mutations | 1 | 0.101 |
| 27 | p.F270S | TP53_GOF_Mutations | 1 | 0.039 |
| 28 | p.G245V | TP53_GOF_Mutations | 1 | 0.062 |
| 29 | p.G266E | TP53_GOF_Mutations | 1 | 0.051 |
| 30 | p.G266R | TP53_GOF_Mutations | 1 | 0.378 |
| 31 | p.G279E | TP53_GOF_Mutations | 1 | 0.371 |
| 32 | p.H179N | TP53_GOF_Mutations | 1 | 0.057 |
| 33 | p.H214R | TP53_GOF_Mutations | 1 | 0.463 |
| 34 | p.H233D | TP53_GOF_Mutations | 1 | 0.283 |
| 35 | p.K139N | TP53_GOF_Mutations | 1 | 0.047 |
| 36 | p.L194F | TP53_GOF_Mutations | 1 | 0.055 |
| 37 | p.L194H | TP53_GOF_Mutations | 1 | 0.389 |
| 38 | p.L194R | TP53_GOF_Mutations | 1 | 0.854 |
| 39 | p.M237L | TP53_GOF_Mutations | 1 | 0.278 |
| 40 | p.M246V | TP53_GOF_Mutations | 1 | 0.237 |
| 41 | p.P128Rfs*38 | TP53_GOF_Mutations | 1 | 0.282 |
| 42 | p.P152L | TP53_GOF_Mutations | 1 | 0.129 |
| 43 | p.P278S | TP53_GOF_Mutations | 1 | 0.015 |
| 44 | p.P295Hfs*38 | TP53_GOF_Mutations | 1 | 0.305 |
| 45 | p.Q144* | TP53_GOF_Mutations | 1 | 0.032 |
| 46 | p.R110L | TP53_GOF_Mutations | 1 | 0.069 |
| 47 | p.R181C | TP53_GOF_Mutations | 1 | 0.086 |
| 48 | p.R280T | TP53_GOF_Mutations | 1 | 0.130 |
| 49 | p.R282G | TP53_GOF_Mutations | 1 | 0.651 |
| 50 | p.S215I | TP53_GOF_Mutations | 1 | 0.376 |
| 51 | p.T284P | TP53_GOF_Mutations | 1 | 0.140 |
| 52 | p.V173L | TP53_GOF_Mutations | 1 | 0.299 |
| 53 | p.V173M | TP53_GOF_Mutations | 1 | 0.396 |
| 54 | p.V274F | TP53_GOF_Mutations | 1 | 0.547 |
| 55 | p.Y205C | TP53_GOF_Mutations | 1 | 0.227 |
| 56 | p.Y234C | TP53_GOF_Mutations | 1 | 0.017 |
| 57 | p.Y234H | TP53_GOF_Mutations | 1 | 0.335 |
| 58 | p.Q192* | TP53_Other_Mutations | 7 | 0.210 |
| 59 | p.I195T | TP53_Other_Mutations | 5 | 0.261 |
| 60 | p.Q331* | TP53_Other_Mutations | 4 | 0.382 |
| 61 | p.C135F | TP53_Other_Mutations | 3 | 0.255 |
| 62 | p.C242F | TP53_Other_Mutations | 3 | 0.539 |
| 63 | p.R196* | TP53_Other_Mutations | 3 | 0.266 |
| 64 | p.R342* | TP53_Other_Mutations | 3 | 0.111 |
| 65 | p.C135Y | TP53_Other_Mutations | 2 | 0.283 |
| 66 | p.E298* | TP53_Other_Mutations | 2 | 0.308 |
| 67 | p.E339* | TP53_Other_Mutations | 2 | 0.150 |
| 68 | p.G105C | TP53_Other_Mutations | 2 | 0.270 |
| 69 | p.G244D | TP53_Other_Mutations | 2 | 0.130 |
| 70 | p.G266V | TP53_Other_Mutations | 2 | 0.263 |
| 71 | p.G334V | TP53_Other_Mutations | 2 | 0.224 |
| 72 | p.H193R | TP53_Other_Mutations | 2 | 0.296 |
| 73 | p.K132N | TP53_Other_Mutations | 2 | 0.244 |
| 74 | p.N131Kfs*18 | TP53_Other_Mutations | 2 | 0.146 |
| 75 | p.P142Lfs*28 | TP53_Other_Mutations | 2 | 0.473 |
| 76 | p.P278S | TP53_Other_Mutations | 2 | 0.483 |
| 77 | p.Q136* | TP53_Other_Mutations | 2 | 0.317 |
| 78 | p.Q136E | TP53_Other_Mutations | 2 | 0.321 |
| 79 | p.Q144* | TP53_Other_Mutations | 2 | 0.231 |
| 80 | p.R158L | TP53_Other_Mutations | 2 | 0.066 |
| 81 | p.R248L | TP53_Other_Mutations | 2 | 0.240 |
| 82 | p.R337C | TP53_Other_Mutations | 2 | 0.387 |
| 83 | p.R65Efs*58 | TP53_Other_Mutations | 2 | 0.088 |
| 84 | p.V203L | TP53_Other_Mutations | 2 | 0.313 |
| 85 | p.Y234D | TP53_Other_Mutations | 2 | 0.343 |
| 86 | p.A159P | TP53_Other_Mutations | 1 | 0.606 |
| 87 | p.A159Sfs*5 | TP53_Other_Mutations | 1 | 0.033 |
| 88 | p.A161D | TP53_Other_Mutations | 1 | 0.468 |
| 89 | p.A161T | TP53_Other_Mutations | 1 | 0.050 |
| 90 | p.A347D | TP53_Other_Mutations | 1 | 0.179 |
| 91 | p.A347Pfs*23 | TP53_Other_Mutations | 1 | 0.053 |
| 92 | p.A63Vfs*60 | TP53_Other_Mutations | 1 | 0.280 |
| 93 | p.A86Vfs*55 | TP53_Other_Mutations | 1 | 0.550 |
| 94 | p.A88Gfs*32 | TP53_Other_Mutations | 1 | 0.027 |
| 95 | p.A88Pfs*35 | TP53_Other_Mutations | 1 | 0.296 |
| 96 | p.C135G | TP53_Other_Mutations | 1 | 0.653 |
| 97 | p.C141Y | TP53_Other_Mutations | 1 | 0.200 |
| 98 | p.C176G | TP53_Other_Mutations | 1 | 0.189 |
| 99 | p.C176W | TP53_Other_Mutations | 1 | 0.309 |
| 100 | p.C182Wfs*22 | TP53_Other_Mutations | 1 | 0.021 |
| 101 | p.C238S | TP53_Other_Mutations | 1 | 0.154 |
| 102 | p.C242L | TP53_Other_Mutations | 1 | 0.217 |
| 103 | p.C242R | TP53_Other_Mutations | 1 | 0.025 |
| 104 | p.C242S | TP53_Other_Mutations | 1 | 0.068 |
| 105 | p.C242_M243delinsW | TP53_Other_Mutations | 1 | 0.071 |
| 106 | p.C275G | TP53_Other_Mutations | 1 | 0.092 |
| 107 | p.D184Afs*62 | TP53_Other_Mutations | 1 | 0.128 |
| 108 | p.D228Vfs*18 | TP53_Other_Mutations | 1 | 0.054 |
| 109 | p.D41Vfs*2 | TP53_Other_Mutations | 1 | 0.190 |
| 110 | p.E171* | TP53_Other_Mutations | 1 | 0.051 |
| 111 | p.E204* | TP53_Other_Mutations | 1 | 0.585 |
| 112 | p.E221* | TP53_Other_Mutations | 1 | 0.187 |
| 113 | p.E258G | TP53_Other_Mutations | 1 | 0.067 |
| 114 | p.E271_V272delinsAL | TP53_Other_Mutations | 1 | 0.037 |
| 115 | p.E285* | TP53_Other_Mutations | 1 | 0.271 |
| 116 | p.E285Gfs*60 | TP53_Other_Mutations | 1 | 0.170 |
| 117 | p.E285K | TP53_Other_Mutations | 1 | 0.320 |
| 118 | p.E286K | TP53_Other_Mutations | 1 | 0.335 |
| 119 | p.E336* | TP53_Other_Mutations | 1 | 0.046 |
| 120 | p.E388Dfs*28 | TP53_Other_Mutations | 1 | 0.022 |
| 121 | p.E51* | TP53_Other_Mutations | 1 | 0.112 |
| 122 | p.E62Gfs*87 | TP53_Other_Mutations | 1 | 0.028 |
| 123 | p.F113V | TP53_Other_Mutations | 1 | 0.289 |
| 124 | p.F212Mfs*27 | TP53_Other_Mutations | 1 | 0.264 |
| 125 | p.F270V | TP53_Other_Mutations | 1 | 0.031 |
| 126 | p.G105S | TP53_Other_Mutations | 1 | 0.428 |
| 127 | p.G154V | TP53_Other_Mutations | 1 | 0.368 |
| 128 | p.G199* | TP53_Other_Mutations | 1 | 0.339 |
| 129 | p.G244C | TP53_Other_Mutations | 1 | 0.150 |
| 130 | p.G266* | TP53_Other_Mutations | 1 | 0.451 |
| 131 | p.G266Dfs*79 | TP53_Other_Mutations | 1 | 0.379 |
| 132 | p.G266_R273dup | TP53_Other_Mutations | 1 | 0.046 |
| 133 | p.G334_R335del | TP53_Other_Mutations | 1 | 0.090 |
| 134 | p.H115Ifs*8 | TP53_Other_Mutations | 1 | 0.246 |
| 135 | p.H193Y | TP53_Other_Mutations | 1 | 0.484 |
| 136 | p.H214Y | TP53_Other_Mutations | 1 | 0.274 |
| 137 | p.I251S | TP53_Other_Mutations | 1 | 0.226 |
| 138 | p.I255del | TP53_Other_Mutations | 1 | 0.343 |
| 139 | p.I332N | TP53_Other_Mutations | 1 | 0.391 |
| 140 | p.K120Sfs*3 | TP53_Other_Mutations | 1 | 0.132 |
| 141 | p.K382Nfs*40 | TP53_Other_Mutations | 1 | 0.221 |
| 142 | p.L201* | TP53_Other_Mutations | 1 | 0.187 |
| 143 | p.L206Sfs*41 | TP53_Other_Mutations | 1 | 0.124 |
| 144 | p.L257V | TP53_Other_Mutations | 1 | 0.505 |
| 145 | p.L330Ffs*15 | TP53_Other_Mutations | 1 | 0.273 |
| 146 | p.M243Ifs*6 | TP53_Other_Mutations | 1 | 0.463 |
| 147 | p.N210Tfs*37 | TP53_Other_Mutations | 1 | 0.118 |
| 148 | p.P128Lfs*42 | TP53_Other_Mutations | 1 | 0.256 |
| 149 | p.P128Wfs*33 | TP53_Other_Mutations | 1 | 0.047 |
| 150 | p.P142Vfs*28 | TP53_Other_Mutations | 1 | 0.279 |
| 151 | p.P152Rfs*18 | TP53_Other_Mutations | 1 | 0.047 |
| 152 | p.P278A | TP53_Other_Mutations | 1 | 0.197 |
| 153 | p.P278L | TP53_Other_Mutations | 1 | 0.351 |
| 154 | p.P27Lfs*17 | TP53_Other_Mutations | 1 | 0.261 |
| 155 | p.P47Rfs*76 | TP53_Other_Mutations | 1 | 0.326 |
| 156 | p.P72Nfs*50 | TP53_Other_Mutations | 1 | 0.076 |
| 157 | p.P82Rfs*41 | TP53_Other_Mutations | 1 | 0.480 |
| 158 | p.Q144P | TP53_Other_Mutations | 1 | 0.316 |
| 159 | p.Q165* | TP53_Other_Mutations | 1 | 0.360 |
| 160 | p.Q331Rfs*14 | TP53_Other_Mutations | 1 | 0.021 |
| 161 | p.Q52* | TP53_Other_Mutations | 1 | 0.506 |
| 162 | p.R156Afs*14 | TP53_Other_Mutations | 1 | 0.769 |
| 163 | p.R158P | TP53_Other_Mutations | 1 | 0.130 |
| 164 | p.R158Pfs*12 | TP53_Other_Mutations | 1 | 0.211 |
| 165 | p.R158Pfs*9 | TP53_Other_Mutations | 1 | 0.104 |
| 166 | p.R181H | TP53_Other_Mutations | 1 | 0.522 |
| 167 | p.R209Kfs*6 | TP53_Other_Mutations | 1 | 0.172 |
| 168 | p.R213* | TP53_Other_Mutations | 1 | 0.362 |
| 169 | p.R248Lfs*97 | TP53_Other_Mutations | 1 | 0.194 |
| 170 | p.R249Sfs*96 | TP53_Other_Mutations | 1 | 0.167 |
| 171 | p.R280S | TP53_Other_Mutations | 1 | 0.022 |
| 172 | p.R283Afs*62 | TP53_Other_Mutations | 1 | 0.362 |
| 173 | p.R379Ifs*42 | TP53_Other_Mutations | 1 | 0.064 |
| 174 | p.R65* | TP53_Other_Mutations | 1 | 0.328 |
| 175 | p.S215R | TP53_Other_Mutations | 1 | 0.341 |
| 176 | p.S227Lfs*20 | TP53_Other_Mutations | 1 | 0.232 |
| 177 | p.S240G | TP53_Other_Mutations | 1 | 0.047 |
| 178 | p.S241Y | TP53_Other_Mutations | 1 | 0.904 |
| 179 | p.S241del | TP53_Other_Mutations | 1 | 0.325 |
| 180 | p.S315Lfs*30 | TP53_Other_Mutations | 1 | 0.642 |
| 181 | p.S367R | TP53_Other_Mutations | 1 | 0.415 |
| 182 | p.T102Rfs*48 | TP53_Other_Mutations | 1 | 0.047 |
| 183 | p.V172F | TP53_Other_Mutations | 1 | 0.292 |
| 184 | p.V172G | TP53_Other_Mutations | 1 | 0.170 |
| 185 | p.V216L | TP53_Other_Mutations | 1 | 0.218 |
| 186 | p.V272M | TP53_Other_Mutations | 1 | 0.208 |
| 187 | p.V73Rfs*76 | TP53_Other_Mutations | 1 | 0.583 |
| 188 | p.W53Mfs*4 | TP53_Other_Mutations | 1 | 0.183 |
| 189 | p.Y126H | TP53_Other_Mutations | 1 | 0.454 |
| 190 | p.Y126S | TP53_Other_Mutations | 1 | 0.258 |
| 191 | p.Y205* | TP53_Other_Mutations | 1 | 0.128 |
| 192 | p.Y220Mfs*27 | TP53_Other_Mutations | 1 | 0.081 |
| 193 | p.Y234N | TP53_Other_Mutations | 1 | 0.620 |
| 194 | p.Y236C | TP53_Other_Mutations | 1 | 0.172 |
| 195 | p.Y236H | TP53_Other_Mutations | 1 | 0.143 |
